# Supplementary material for: Alcohol inhibits the metabolism of dimethyl fumarate to the active metabolite responsible for decreasing relapse frequency in the treatment of multiple sclerosis
Source: PLoS One. 2022 Nov 28;17(11):e0278111. doi: 10.1371/journal.pone.0278111 (PMC9704628; doi:10.1371/journal.pone.0278111)
Supplement: S1 Fig — (PDF) [file pone.0278111.s001.pdf]

Fig 2. The Formation of MMF in CES1, CES2, and HIM. DMF was incubated for increasing time periods (0, 5, 10, 20, and 30 minutes) in human recombinant CES1 and CES2, and HIM. The concentration of MMF at the end of each incubation was determined by LC-MS/MS. Only CES1 hydrolyzed DMF to its active MMF metabolite.

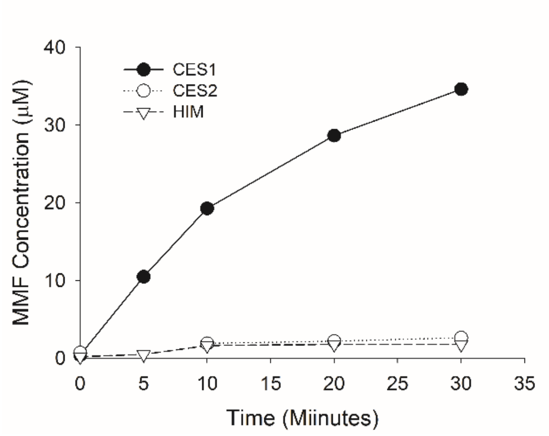

Plot of the monomethyl fumarate (MMF) concentration in µM versus the incubation time (minutes) of 50 µM dimethyl fumarate (DMF) in human recombinant carboxylesterase-1 (CES1) and carboxylesterase-2 (CES2) enzymes, and in human intestinal microsomes (HIM).
Data

	µM of Monomethyl Fumarate Formed		
Time	CES1	CES2	HIM
0	0.41	0.70	0.18
5	10.5		0.50
10	19.3	1.9	1.6
20	28.7	2.2	1.8
30	34.7	2.6	1.7

This is a qualitative assessment (only one concentration per time point) to determine the predominant hydrolysis pathway of DMF.
