# Supplementary material for: Alcohol inhibits the metabolism of dimethyl fumarate to the active metabolite responsible for decreasing relapse frequency in the treatment of multiple sclerosis
Source: PLoS One. 2022 Nov 28;17(11):e0278111. doi: 10.1371/journal.pone.0278111 (PMC9704628; doi:10.1371/journal.pone.0278111)
Supplement: S2 Fig — (PDF) [file pone.0278111.s002.pdf]

Fig 3. The Concentration-Dependent Inhibition of CES1 Catalyzed Hydrolysis by Alcohol. DMF (50 µM) was incubated for 30 minutes in recombinant human CES1 containing increasing concentrations of ethanol (10, 25, 50, 100, and 200 mM). The concentration of MMF at the end of the 30-minute incubation was determined by LC-MS/MS. The estimated IC_{50} was 19.7 mM, which is equivalent to an ethanol concentration of 0.091 g/dL (legal limit for driving under the influence is 0.08 g/dL for point of reference).

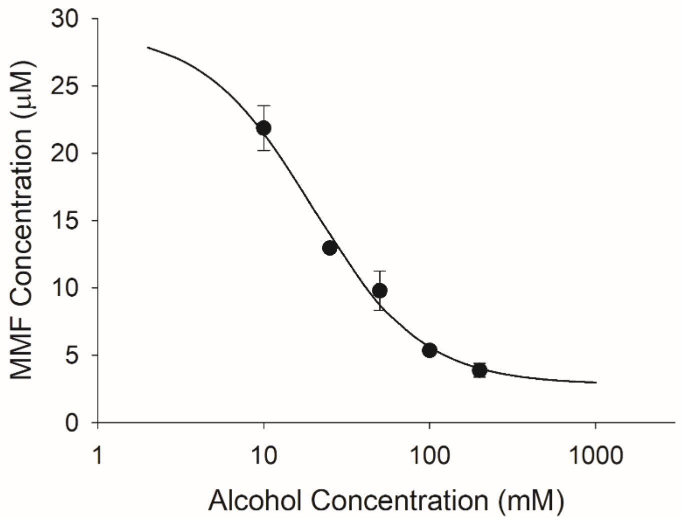

Plot of the MMF concentration in µM versus the alcohol concentration (mM) in human recombinant CES1. The initial DMF concentration is 50 µM and the incubation time is 30 minutes.

Data

	µM of Monomethyl Fumarate Formed				
Alcohol (mM)	Run 1	Run 2	Run 3	Mean	SD
0	28.3	31.2	27.5	29.0	2.0
10	20.6	23.8	21.2	21.9	1.7
25	13.1	13.0	12.8	13.0	0.2
50	9.5	8.5	11.1	9.8	1.3
100	5.4	5.3	5.4	5.4	0.1
200	4.3	3.3	4.1	3.9	0.5
