# Supplementary material for: Alcohol inhibits the metabolism of dimethyl fumarate to the active metabolite responsible for decreasing relapse frequency in the treatment of multiple sclerosis
Source: PLoS One. 2022 Nov 28;17(11):e0278111. doi: 10.1371/journal.pone.0278111 (PMC9704628; doi:10.1371/journal.pone.0278111)
Supplement: S3 Fig — (PDF) [file pone.0278111.s003.pdf]

Fig 4. Hydrolysis of DMF in CES1 with Alcohol Produces Unknown Metabolite(s). DMF (50 µM) was incubated in human recombinant CES1 for 60 minutes and the total amount of DMF and MMF remaining at the end of the incubation was quantified. Compared to the Control, Alcohol (200 mM) resulted in a decrease in the formation of MMF. However, DMF in the incubation containing alcohol was lower than in the Control indicating that hydrolysis products other than MMF were formed. Both the DMF and MMF concentrations were statistically different, with $p<0.05$ between the Control and Alcohol group.

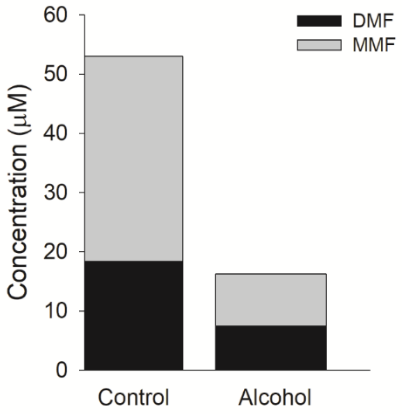

Stacked bar plot of the DMF and MMF concentration in µM after incubating 50 µM of DMF for 30 minutes in recombinant CES1 containing no alcohol (Control) and containing 200 mM of alcohol.

Data

	Run 1	Run 2	Run 3	Mean	SD
Control					
DMF	18.1	16.8	20.3	18.4	1.8
MMF	34.1	34.6	35.2	34.6	0.6
Alcohol					
DMF	8.4	6.5	7.4	7.4	0.9
MMF	9.1	8.5	8.8	8.8	0.4
