# Supplementary material for: Alcohol inhibits the metabolism of dimethyl fumarate to the active metabolite responsible for decreasing relapse frequency in the treatment of multiple sclerosis
Source: PLoS One. 2022 Nov 28;17(11):e0278111. doi: 10.1371/journal.pone.0278111 (PMC9704628; doi:10.1371/journal.pone.0278111)
Supplement: S4 Fig — (PDF) [file pone.0278111.s004.pdf]

Fig 5. DMF Plasma Concentrations in Control and Alcohol group. Each time point represents the mean plasma concentration from three mice with the bars indicating the standard deviation. All concentration-time points were statistically different between the Control and Alcohol group ($p < 0.05$ with a Benjamini-Hochberg Procedure for multiple comparisons).

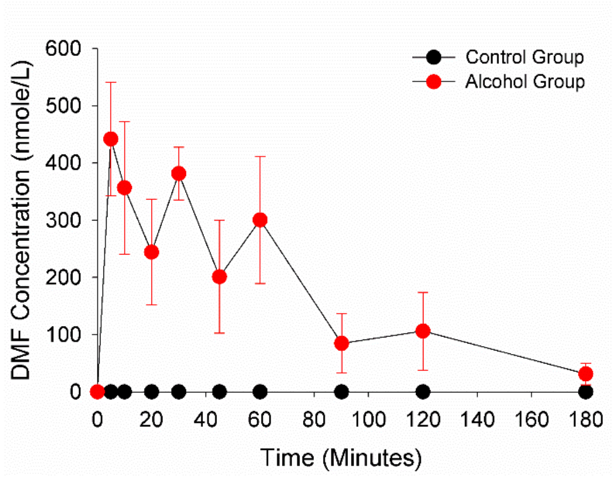

Plasma DMF concentration-time profile (nmole/L versus time in minutes) in two groups of mice, the Control group received DMF 100 mg/kg, and the alcohol group received 3 g/kg alcohol prior to the 100 mg/kg DMF dose.

Data

	Time	Mouse 1	Mouse 2	Mouse 3	Mean	SD	
Control							
	0	0	0	0			
	5	0	0	0			
	10	0	0	0			
	20	0	0	0			
	30	0	0	0			
	45	0	0	0			
	60	0	0	0			
	90	0	0	0			
	120	0	0	0			
	180	0	0	0			
Alcohol							
	0	0	0	0			
	5	432	546	349	442	99	
	10	474	355	242	357	116	
	20	228	161	343	244	92	
	30	347	364	433	381	46	
	45	123	168	312	201	99	
	60	390	336	176	300	111	
	90	35	81	138	85	52	
	120	98	178	43	106	68	
	180	13	51	30	31	19	
