# Supplementary material for: Alcohol inhibits the metabolism of dimethyl fumarate to the active metabolite responsible for decreasing relapse frequency in the treatment of multiple sclerosis
Source: PLoS One. 2022 Nov 28;17(11):e0278111. doi: 10.1371/journal.pone.0278111 (PMC9704628; doi:10.1371/journal.pone.0278111)
Supplement: S5 Fig — (PDF) [file pone.0278111.s005.pdf]

Fig 6. MMF Plasma Concentrations in Control and Alcohol Group. Each time point represents the mean plasma concentration from three mice with the bars indicating the standard deviation. An asterisk beside the concentration-time point indicates the mean concentration difference between the Control and Alcohol group is statistically different ($p < 0.05$ with a Benjamini-Hochberg Procedure for multiple comparisons)

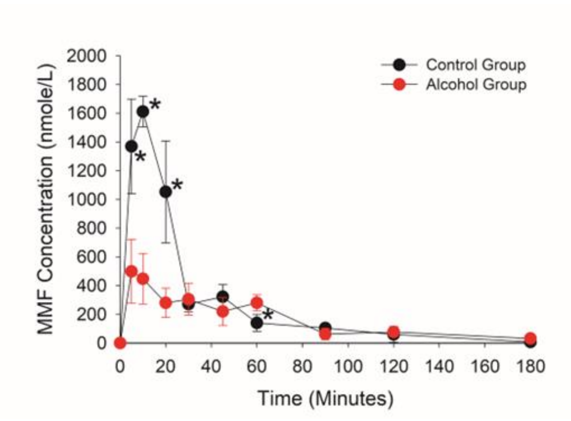

.

Plasma MMF concentration-time profile (nmole/L versus time in minutes) in two groups of mice, the Control group received DMF 100 mg/kg, and the alcohol group received 3 g/kg alcohol prior to the 100 mg/kg DMF dose.

	Time	Mouse 1	Mouse 2	Mouse 3	Mean	SD	
Control							
	0	0	0	0			
	5	1062	1330	1716	1369	329	
	10	1533	1567	1733	1611	107	
	20	1065	692	1400	1053	354	
	30	239	334	239	271	55	
	45	360	380	226	322	84	
	60	95	118	206	140	59	
	90	114	124	76	105	25	
	120	27	30	124	60	55	
	180	7	0	0	2	4	
Alcohol							
	0	0	0	0			
	5	275	502	720	499	222	
	10	610	470	261	447	175	
	20	379	284	177	280	101	
	30	416	197	300	304	110	
	45	309	116	235	220	98	
	60	274	226	339	280	56	
	90	100	21	66	62	40	
	120	50	69	112	77	32	
	180	13	25	56	31	22	
