# Supplementary material for: Alcohol inhibits the metabolism of dimethyl fumarate to the active metabolite responsible for decreasing relapse frequency in the treatment of multiple sclerosis
Source: PLoS One. 2022 Nov 28;17(11):e0278111. doi: 10.1371/journal.pone.0278111 (PMC9704628; doi:10.1371/journal.pone.0278111)
Supplement: S6 Fig — (PDF) [file pone.0278111.s006.pdf]

Fig 7. The Brain Tissue Concentrations of DMF and MMF. The brain tissue concentrations are expressed as the nmole of DMF and MMF per Kg of brain tissue (mean ± standard deviation). An asterisk beside the concentration-time point indicates the mean concentration difference between the Control and Alcohol group is statistically different ($p < 0.05$ with a Benjamini-Hochberg Procedure for multiple comparisons).

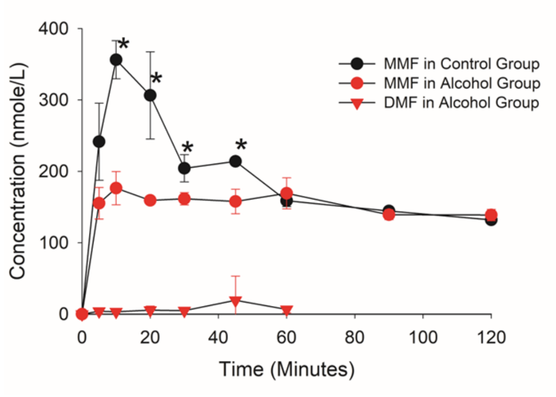

Brain MMF and DMF concentration-time profiles (nmole/g versus time in minutes) in two groups of mice, the Control group received DMF 100 mg/kg, and the alcohol group received 3 g/kg alcohol prior to the 100 mg/kg DMF dose.

MMF Brain Concentrations versus Time						
	Time	Mouse 1	Mouse 2	Mouse 3	Mean	SD
Control						
	0	0	0	0		
	5	180	260	283	241	54
	10	328	360	381	356	27
	20	289	256	374	306	61
	30	189	198	226	204	19
	45	215	208	218	214	5
	60	154	168	155	159	8
	90	149	144	140	144	4
	120	135	135	126	132	5
	180					
Alcohol						
	0	0	0	0		
	5	138	180	148	155	22
	10	150	192	188	176	23
	20	160	155	162	159	4
	30	157	156	171	162	9
	45	141	156	176	158	17
	60	169	191	148	169	22
	90	132	146	139	139	7
	120	135	148	134	139	8
	180					

DMF Brain Concentrations versus Time						
	Time	Mouse 1	Mouse 2	Mouse 3	Mean	SD
Alcohol						
	0	0	0	0		
	5	6	5	0	4	3
	10	4	2	4	3	1
	20	0	4	10	5	5
	30	4	0	9	4	4
	45	0	1	53	18	30
	60	2	6	11	6	4
