# Supplementary material for: Alcohol inhibits the metabolism of dimethyl fumarate to the active metabolite responsible for decreasing relapse frequency in the treatment of multiple sclerosis
Source: PLoS One. 2022 Nov 28;17(11):e0278111. doi: 10.1371/journal.pone.0278111 (PMC9704628; doi:10.1371/journal.pone.0278111)
Supplement: Attachment — Submitted filename: PONE - Reviewer Comments - Authors Response.docx [file pone.0278111.s007.docx]

PONE-D-22-21279

ACADEMIC EDITOR:

The title uses the word patients, but no human studies have been performed. It needs to be changed. Also include the previous experiments with dimethyl fumarate in the Introduction section. Statistical analysis needs to be re-validated. 

The original title, “Alcohol Consumption may Decrease the effectiveness of Dimethyl Fumarate for Preventing Relapses in Patients with Multiple Sclerosis” has been revised to, “Alcohol Inhibits the Metabolism of Dimethyl Fumarate to the Active Metabolite Responsible for Decreasing Relapse Frequency in the Treatment of Multiple Sclerosis”.

Unclear what previous experiments you are referring to that should be put in the introduction. All the experiments described in this paper are original work that has not been published and cannot be included in the introduction section, which would precede the description of how they were done in the Methods section.

We have revised the manuscript to include a basic statistical analysis of the results of the incubation study in the recombinant enzyme (Figure 3), and the concentration-time data from the mouse study (Figures 5, 6, and 7). A description of the statistics has been added to the Methods section. The figures have been revised to include an asterisk by the concentration-time points that are statistically different.

**Comments to the Author**

1. Is the manuscript technically sound, and do the data support the conclusions?

The manuscript must describe a technically sound piece of scientific research with data that supports the conclusions. Experiments must have been conducted rigorously, with appropriate controls, replication, and sample sizes. The conclusions must be drawn appropriately based on the data presented.

Reviewer #1: Yes

Reviewer #2: Yes

2. Has the statistical analysis been performed appropriately and rigorously?

Reviewer #1: Yes

Reviewer #2: Yes

3. Have the authors made all data underlying the findings in their manuscript fully available?

The [PLOS Data policy](http://www.plosone.org/static/policies.action#sharing) requires authors to make all data underlying the findings described in their manuscript fully available without restriction, with rare exception (please refer to the Data Availability Statement in the manuscript PDF file). The data should be provided as part of the manuscript or its supporting information, or deposited to a public repository. For example, in addition to summary statistics, the data points behind means, medians and variance measures should be available. If there are restrictions on publicly sharing data—e.g. participant privacy or use of data from a third party—those must be specified.

Reviewer #1: Yes

Reviewer #2: Yes

4. Is the manuscript presented in an intelligible fashion and written in standard English?

PLOS ONE does not copyedit accepted manuscripts, so the language in submitted articles must be clear, correct, and unambiguous. Any typographical or grammatical errors should be corrected at revision, so please note any specific errors here.

Reviewer #1: Yes

Reviewer #2: Yes

5. Review Comments to the Author

Please use the space provided to explain your answers to the questions above. You may also include additional comments for the author, including concerns about dual publication, research ethics, or publication ethics. (Please upload your review as an attachment if it exceeds 20,000 characters)

Reviewer #1: Title of manuscript “Alcohol Consumption may Decrease the Effectiveness of Dimethyl Fumarate for Preventing Relapses in Patients with Multiple Sclerosis” is a good draft.

Comments:
1. There are large number of articles already in literature about “Dimethyl fumarate for multiple sclerosis”
Ref : https://www.cochranelibrary.com/cdsr/doi/10.1002/14651858.CD011076.pub2/full
https://www.tandfonline.com/doi/abs/10.1517/14740338.2015.977251, What is the novelty in this study?

The novelty of this study is that it is the first to report that alcohol inhibits the formation of the active metabolite, monomethyl fumarate from dimethyl fumarate leading to a reduction in exposure to the active metabolite and quantifiable concentrations of dimethyl fumarate in the plasma. If this interaction between alcohol and dimethyl fumarate is confirmed in a human study, it would most likely require that the interaction be included in the FDA prescribing information.

2. Provide the clear data, effect of dimethyl fumarate for Multiple Sclerosis.

The effect of dimethyl fumarate on multiple sclerosis is elaborated in reference 20. The present study is a pharmacokinetic study, and there is no data presented on the effect of dimethyl fumarate for the treatment of multiple sclerosis.

3. Abbreviation should mention at their first time appearance in the manuscript. Like DMF.

Yes, we agree and have revised it accordingly.

4. Recommended to design the few more 2-3 tables for the manuscript based on the discussion in the manuscript.

Unclear what the reviewer is proposing to be included in these tables.

5. What is the comparative effectiveness of the DMF and MMF? Provide the date in table format.

This is unknown as DMF has never been clinically evaluated in the treatment of multiple sclerosis since it is impossible to achieve quantifiable levels in humans. It is theorized that DMF would have an effect on multiple sclerosis if it achieved significant plasma concentrations based on its proposed effect on the Nrf2 pathway demonstrated in in vitro studies; however, this is hypothetical. This study only demonstrates that alcohol drastically changes the disposition of DMF. How these changes other than the decrease in active metabolite alter its therapeutic activity are speculative and beyond the scope of this study.

6. Elaborate the supporting information.

Supplemental supporting information documents has been added.

7. Recommended to author to cite the latest publication.

The reviewer would have to be more specific for me to address this issue.

8. Figure 1 is blurr, need to draw the structures.

I think the concatenation in forming the pdf led to a decrease in resolution. For the revision, we assessed and modified all the figures using the PACE program recommended by the publisher.

Reviewer #2: The manuscript entitled Alcohol Consumption may Decrease the Effectiveness of Dimethyl Fumarate for Preventing Relapses in Patients with Multiple Sclerosis is a good piece of research work. Although as a reviewer I have general suggestions and queries. which are appended below:

1. In Introduction section the background about multiple sclerosis and the action of DMF in multiple sclerosis is lacking which is important for readers to understand.

We have added a statement in the introduction on the specific therapeutic effect of DMF in the treatment of multiple sclerosis.

2. Line 55: For hypothesis the support and reference must be sufficiently elaborated in favour of CES1.

Previous support for our hypothesis from the literature does not exist. The data presented in this manuscript in recombinant enzymes and HIM provide the support that DMF is a CES1 substrate and was used to justify conducting the study in mice. The results from the mouse study confirm that it is a CES1 substrate, which is susceptible to inhibition by alcohol, a well-known CES1 enzyme inhibitor.

3. Further, ruling of transesterification needs references.

I am unclear as to what the reviewer means by “ruling”.

4.For In vitro incubation studies please provide the reference as well as mention why the studies are conducted in duplicate not in triplicate?

All the incubation studies were conducted in triplicate except for the initial qualitative study in CES1, CES2, and HIM (Figure 2) in which each time point represents a single incubation. We have revised the text in the manuscript to reflect this and we have also provided a supplementary data file giving the raw data used to construct the figures. It is unclear what the reviewer is asking us to reference.

5. For In vivo studies in mice the details of model followed with protocol is desirable in methods section as well as please mention the reference for dose of 3g/kg alcohol. also, mention the quantity of retro-orbital blood samples for better understanding of readers.

We have added statements to the In Vivo Study in the Es1^e^ Mice section in Methods referencing the Es1^e^ mouse model, and the dose of DMF and alcohol used in the mouse study. The blood volume collected has been added to the text.

6. In results in DMF alcohol study please mention the data in text for the ease.

The data in Table 2 is referenced in the text and we have added the specific data in the text on the comparison of the DMF and MMF AUC in the Control and Alcohol groups from the table.

7. The recommendation in conclusion section regarding alcohol consumption two hours before and after if supported by the result and findings from your work would be appreciated.

We have revised this section of the discussion to better reflect the data from this study by removing the specific time recommendation and simply indicating that consumption of alcohol close to DMF dosing may decrease MMF exposure.

6. PLOS authors have the option to publish the peer review history of their article ([what does this mean?](https://journals.plos.org/plosone/s/editorial-and-peer-review-process#loc-peer-review-history)). If published, this will include your full peer review and any attached files.
